# Can scientific journals benefit from a social media presence? An analysis of online traffic data and author perspectives

**DOI:** 10.1016/j.rpth.2024.102387

**Published:** 2024-03-18

**Authors:** Mouhamed Yazan Abou-Ismail, Dianne E. van der Wal, May Anne Cheong, Andrew Masten, Luke Blount, Megan C. Brown

**Affiliations:** 1 Division of Hematology & Hematologic Malignancies, University of Utah, Salt Lake City, Utah, USA; 2 Australian Red Cross Lifeblood R&D, Sydney, New South Wales, Australia; 3 Department of Haematology, Singapore General Hospital, Singapore; 4 International Society on Thrombosis and Haemostasis, Carrboro, North Carolina, USA; 5 Department of Pediatrics, Emory University, Atlanta, Georgia, USA; 6 Aflac Cancer and Blood Disorders at Children's Healthcare of Atlanta, Atlanta, Georgia, USA

**Keywords:** information dissemination, journal impact factor, social media

Social media platforms offer a unique opportunity for scientific literature to reach a large audience. Rather than requiring an individual to search for the terms that lead to a piece of scientific work, social media allows scientists and journals to actively disseminate their work to a large network. This allows instantaneous promotion of scientific work to a large audience and promotes dissemination, discussion, and collaboration. In 2017, it was estimated that 1% to 5% of the 187 million active users on X (formerly Twitter) were active scientists [1]. Journal engagement in social media is on the rise. A recent study demonstrated that as of early 2022, 25.2% of all journals listed in Web of Science’s 3 major indices had a dedicated presence on X [2]. This represented a substantial percentage increase from just 0.1% in 2007, which continued to grow, reaching 2.9% in 2010, 11.8% in 2015, and 22.8% in 2020. These findings underscore a swiftly and continuously expanding overlap between social media and scholarly communication, highlighting the growing significance of digital platforms in disseminating scientific information.

Social media engagement has been shown to correlate with citation rates, and a single randomized controlled study found that tweeted articles achieved higher change in citation number in 1 year as compared with nontweeted articles [3]. Both Research and Practice in Thrombosis and Haemostasis (RPTH) and Journal of Thrombosis and Haemostasis (JTH), the journals of the International Society on Thrombosis and Haemostasis (ISTH), maintain an active social media presence. Both journals actively promote every published article through social media channels [4,5]. ISTH has recognized the importance of social media for dissemination of science, starting the X accounts for both journals in 2016. Additionally, JTH started a presence on LinkedIn in 2022. While there is increasing data supporting the role of social media in scientific publication, there remains a paucity of data related to the impact on authors. This study aims to investigate author perspectives on the use of social media for promotion of their unique scientific work, as well as the amount of traffic generated to the RPTH and JTH websites via social media.

In May 2023, we created a survey using Alchemer that was emailed to all corresponding authors who have previously published work with RPTH or JTH. It was designed to be brief (approximately 5 minutes). The survey contained multiple choice and Likert scale questions to determine the effect the journals’ social media presence had on the authors’ choice of publications. The survey was then emailed to a group of corresponding authors from RPTH and JTH. A follow-up email was sent a week later. The email contained a brief description of the goals of the survey as well as some information on the benefits of a paper being posted on social media to increase citation numbers. Survey answers were collected through Alchemer. The online traffic to the RPTH and JTH websites was measured by Elsevier using Adobe Analytics. The data measured referring domains for all visits to the websites from January 1, 2023, through December 31, 2023.

Out of 1195 survey recipients, a total of 115 completed the survey (response rate of 9.6%). The full survey results are shown in the Supplementary material. The social media platform most actively used by our respondents was X (55.8%), followed by LinkedIn (46%) and Facebook (31.9%). Out of all respondents, 54.8% and 47% followed the X accounts of JTH and RPTH, respectively. The frequency at which authors read posts made by RPTH (Figure 1A) and JTH (Supplementary material) was similar for both journals, where the majority read posts by both journals to some extent, and 36% did not read any posts by either journal. X was the platform authors most strongly preferred to have their papers promoted at (Figure 1B).

**Figure 1:**
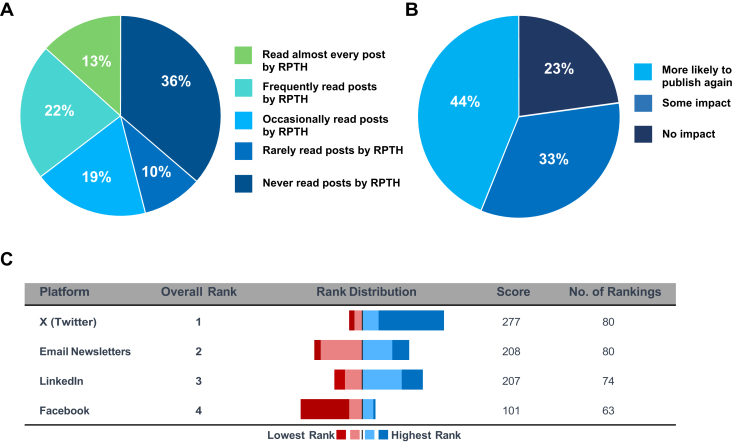
Respondent frequency of reading social media posts by Research and Practice in Thrombosis and Haemostasis (A), likelihood of publishing again with a journal that shares publications on its social media platforms (B), and preferred social media platform for promoting publications (C). RPTH, Research and Practice in Thrombosis and Haemostasis.

The majority of respondents (43.9%) reported they would more likely publish again with a journal that shares their work on its social media platforms, and 33.3% were somewhat more likely to publish again, while only 22.8% were not impacted (Figure 1C). Of interest, the majority (60%) of respondents who did not use social media at that time were interested in being contacted if their paper had high engagement on X. The majority (61%) preferred to write their own messages to be used in social media promotion.

Out of all the total traffic to the RPTH website between January 1, 2023, and December 31, 2023, a significant 14.6% was directed from social media platforms (Table), with the highest being X (11%), followed by Facebook (1.9%) and LinkedIn (1.7%). The numbers may also be higher than reported, as they do not account for users who copy the web address or title of an article from a social media post into a web browser instead of directly clicking a link from a social media post. Compared with JTH, the RPTH website had a higher overall percentage of referrals from social media (6.7% vs 14.6%) but a lower absolute number of visits (12,267 vs 8785 visits; Table). Despite the lower overall social media referral percentage to JTH compared with RPTH, JTH still had a slightly higher percentage of LinkedIn referrals compared with RPTH (2.1% vs 1.7%), and a larger proportion of its social media referrals originated from LinkedIn. This demonstrates the effect of active journal involvement in a specific platform on generating interest and traffic to the journal articles since JTH has a LinkedIn presence and RPTH does not (at the time of this publication).

**Table:** Sources of traffic to RPTH and JTH websites between January 1, 2023, and December 31, 2023.

Traffic source (January 1-December 31, 2023)	RPTH		JTH	
	Number of visits (total = 60,302)	Percentage of visits	Number of visits (total = 184,283)	Percentage of visits
Elsevier website	17,837	29.6	92,467	50.2
Typed/bookmarked	14,733	24.4	31,548	17.1
Google	13,388	22.2	31,324	17.0
ISTH Society websites				
RPTH Journal website	10,179	16.9	209	0.1
JTH Journal website	429	0.7	31,140	16.9
ISTH main website	751	1.2	5329	2.9
Any social media	8785	14.6	12,267	6.7
X (Twitter)	6636	11.0	7501	4.1
LinkedIn	1022	1.7	3878	2.1
Facebook	1127	1.9	888	0.5

ISTH, International Society on Thrombosis and Haemostasis; JTH, Journal of Thrombosis and Haemostasis; RPTH, Research and Practice in Thrombosis and Haemostasis.

Our findings reveal notable author presence on social media, a majority of whom were active users who read the posts made by RPTH and JTH, suggesting a pervasive interest in following scientific publications on social media platforms (mostly X in May 2023). A significant finding of our survey was that the authors’ decision to publish again with the same journal was notably impacted by the journal’s social media presence and its ability to disseminate their work on social media. This implies a beneficial effect of social media presence of scientific or medical journals on journal selection and author preferences. It also suggests that authors appear to recognize the potential advantages of increased visibility and accessibility that come with social media promotion. This may be due to an implicit understanding of the positive relationship between social media exposure and citation numbers [3], indicating a strategic awareness among authors regarding the promotional value of these platforms.

Our data on journal website traffic generated from social media platforms throughout the year 2023 underscores the instrumental role of social media in enhancing the visibility of journals. The significant proportion of website traffic emanating from X suggests that social media platforms can serve as effective conduits for directing users to journal content. This is also highlighted by the larger amount of traffic generated from LinkedIn toward JTH, which has a LinkedIn presence, compared with RPTH, which currently does not. Journals should recognize the potential of social media not only as a tool for author engagement but also as a means of amplifying the reach of their published work and ultimately the extent of scientific dissemination. Both our survey and traffic data findings can provide insight that can impact journal editorial and marketing strategies by focusing on enhancing social media presence as a means of fostering stronger interest in being a target journal for authors and increasing the visibility of the journal and its publications. This can be achieved by delegating social media management as a unique role, requiring a regular upkeep of the journal’s social media presence, engagement, and dissemination of its publications on social media platforms. Journals should also recognize the changes in usage and focus of different social media platforms, which may require adjustments in social media strategies.

Our findings have several limitations. There may have been a potential selection bias of respondents, as those who participated in the surveys might have been more inclined toward active social media usage, and we did not analyze for different variables among authors. Furthermore, our research is specific to only two journals, which may limit the generalizability of our findings. Future research could explore a broader spectrum of journals and disciplines to ascertain the generalizability of these findings. Additionally, investigating the academic rank or position, demographic variables, and geographic location of authors could enhance our understanding of the patterns in author engagement with social media platforms and shed light on their significance for authors.

In conclusion, our study illuminates the multifaceted benefits of integrating social media into the scholarly publishing landscape. The positive impact on journal visibility, website traffic, and authors’ likelihood to submit with the same journal highlights the need for journals to adopt strategic and targeted social media practices. Ultimately, this may help journals foster stronger interest in being a target for publication, enhance visibility and overall scientific dissemination, and generate awareness of its content and unique field of science.

## Supplementary Material

Survey results
