# Supplementary material for: Can scientific journals benefit from a social media presence? An analysis of online traffic data and author perspectives
Source: Res Pract Thromb Haemost. 2024 Mar 18;8(3):102387. doi: 10.1016/j.rpth.2024.102387 (PMC11015501; doi:10.1016/j.rpth.2024.102387)
Supplement: Survey results [file mmc1.docx]

Report for JTH/RPTH Social Media Survey

Response Counts


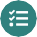


JTH/RPTH Social Media Survey


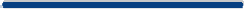
**Completion Rate: 100%**


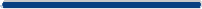
Complete 115

**Totals: 115**

1. Which of the following do you actively use? Check all that apply


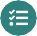


JTH/RPTH Social Media Survey


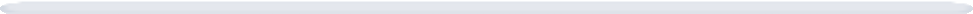

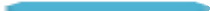


60

50

40

30

Percent

20

10

0

Twitter Facebook LinkedIn None of the Above

**Value Percent Responses**

X (Twitter) 55.8% 63


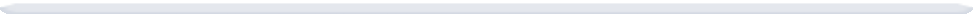

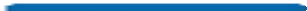

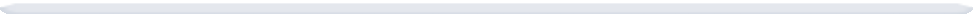

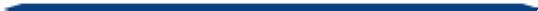


Facebook

31.9%

36

LinkedIn 46.0% 52


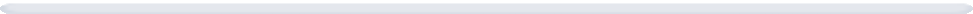

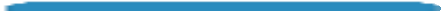


None of the Above

22.1%

25

1. Please rank these platforms by how much you'd like to see your papers promoted on them, with 1 being the highest priority.


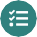


JTH/RPTH Social Media Survey

| **Item** | **Overall Rank** | **Rank Distribution** | **Score** | **No. of Rankings** |
| --- | --- | --- | --- | --- |
|  |  |  |  |  |
| X (Twitter) | 1 |  | 277 | 80 |
|  |  |  |  |  |
|  |  |  |  |  |
| Email Newsletters | 2 |  | 208 | 80 |
|  |  |  |  |  |
|  |  |  |  |  |
| LinkedIn | 3 |  | 207 | 74 |
|  |  |  |  |  |
|  |  |  |  |  |
| Facebook | 4 |  | 101 | 63 |
|  |  |  |  |  |
|  |  |  |  |  |
|  |  |  |  |  |
|  |  | Lowest Rank Highest Rank |  |  |

1. When a journal shares your work on its social media platforms, how much does it positively impact your experience and willingness to publish again with the same journal?


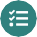


JTH/RPTH Social Media Survey

**23% No impact**


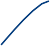

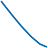


**44% More likely to publish again**

**33% Some impact**

| **Value** | **Percent** | **Responses** |
| --- | --- | --- |
| No impact | 22.8% 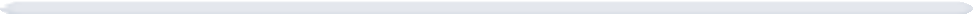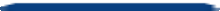 | 26 |
| Some impact | 33.3% 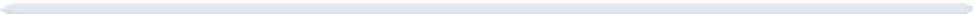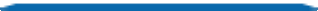 | 38 |
| More likely to publish again | 43.9% 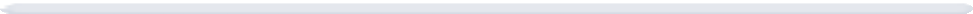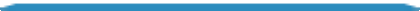 | 50 |
|  |  | **Totals: 114** |

1. Do you follow RPTH and/or JTH on X (Twitter)? Check all that apply.


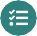


JTH/RPTH Social Media Survey

60

50

40

30

Percent

20

10

0

JTH RPTH Neither Other - Write In (Required)

| **Value** | **Percent** | **Responses** |
| --- | --- | --- |
| JTH | 54.8% 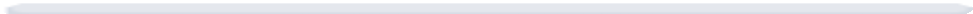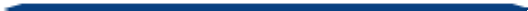 | 63 |
| RPTH | 47.0% 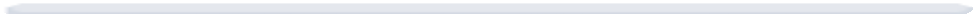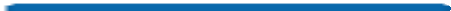 | 54 |
| Neither | 41.7% 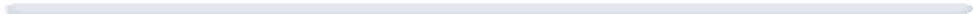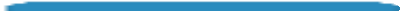 | 48 |
| Other - Write In (Required) | 7.0% 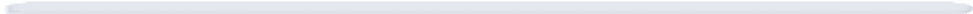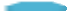 | 8 |

**Other - Write In (Required) Count**

Blood 1

Chest, NEJM, BMJ 1

Haemophilia, Journal of Haemophilia Practice 1

I don't use twitter 1

Thrombosis Research, Lancet Hematology, current opinion in Hematology, SEminars Thrombosis 1 and Hemaostasis

Thrombosis Rsearch 1

WeChat if it is applicable 1

thrombosis research 1

Totals 8

1. How often do you read X (Twitter) posts from JTH?


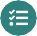


JTH/RPTH Social Media Survey

**12% I read almost every post from JTH**


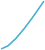


**36% I never read posts from JTH**

**28% I frequently read posts from JTH**

**9% I rarely read posts from JTH**

**15% I occasionally read posts from JTH**

| **Value** | **Percent** | **Responses** |
| --- | --- | --- |
| I read almost every post from JTH | 12.3% 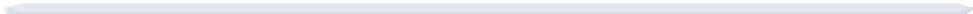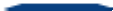 | 14 |
| I frequently read posts from JTH | 28.1% 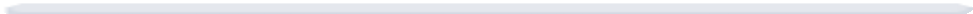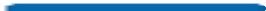 | 32 |
| I occasionally read posts from JTH | 14.9% 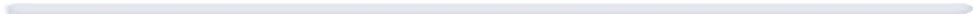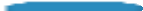 | 17 |
| I rarely read posts from JTH | 8.8% 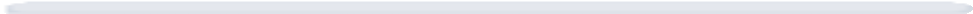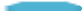 | 10 |
| I never read posts from JTH | 36.0% 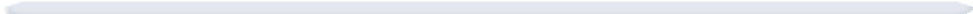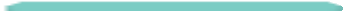 | 41 |
|  |  | **Totals: 114** |

1. How often do you read X (Twitter) posts from RPTH?


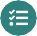


JTH/RPTH Social Media Survey

**13% I read almost every post from RPTH**


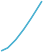


**36% I never read posts from RPTH**

**22% I frequently read posts from RPTH**

**10% I rarely read posts from RPTH**

**19% I occasionally read posts from RPTH**

| **Value** | **Percent** | **Responses** |
| --- | --- | --- |
| I read almost every post from RPTH | 13.3% 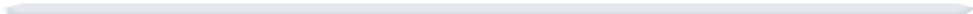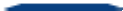 | 15 |
| I frequently read posts from RPTH | 22.1% 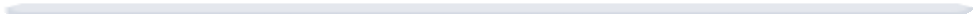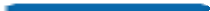 | 25 |
| I occasionally read posts from RPTH | 18.6% 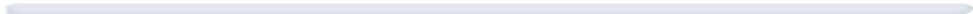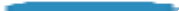 | 21 |
| I rarely read posts from RPTH | 9.7% 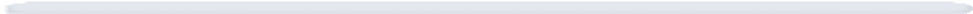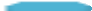 | 11 |
| I never read posts from RPTH | 36.3% 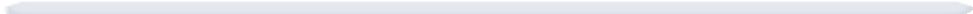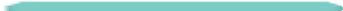 | 41 |
|  |  | **Totals: 113** |

1. IF YOU ARE NOT ON SOCIAL MEDIA- Would you like to be contacted when there is high engagement on your paper on X (Twitter)?


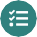


JTH/RPTH Social Media Survey

**10% Other - Write In (Required)**

**31% No**

**60% Yes**

| **Value** | **Percent** | **Responses** |
| --- | --- | --- |
| Yes | 59.8% 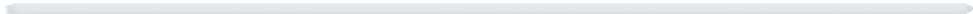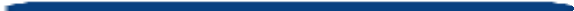 | 49 |
| No | 30.5% 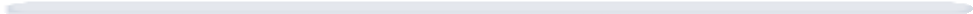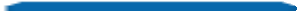 | 25 |
| Other - Write In (Required) | 9.8% 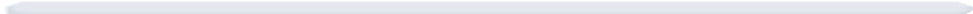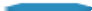 | 8 |
|  |  | **Totals: 82** |

**Other - Write In (Required) Count**

Already on twitter 1

I am already on social media 1

I am on social media 1

I'm on social media 1

N/A 1

NA 1

Yes, but when it concerns LinkedIn 1

not applicable 1

Totals 8

1. Are you interested in writing the messages the journals use to promote your paper?


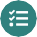


JTH/RPTH Social Media Survey

**4% Other - Write In**

**(Required)**

**35% No**

**61% Yes**

| **Value** | **Percent** | **Responses** |
| --- | --- | --- |
| Yes | 61.1% 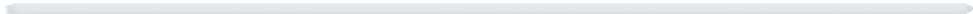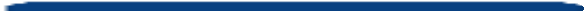 | 69 |
| No | 34.5% 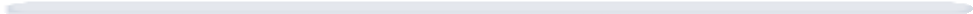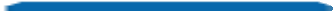 | 39 |
| Other - Write In (Required) | 4.4% 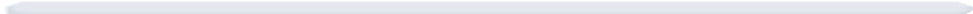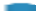 | 5 |
|  |  | **Totals: 113** |

**Other - Write In (Required) Count**

I think the journal knows best and probably has more experience in writing catchy messages 1

Maybe - but I don't have the time I think!! 1

Maybe? I might be interested in editing a suggested message as opposed to composing one. 1

Yes, but very much need help with this part. I like the blurbs that make me want to click, even if 1 not all the detail is there. THose heavy in detail make it less likely I'll finish to see the punchline.

happy to participate 1

Totals 5
